# Diverse Roles of TgMIC1/4/6 in the *Toxoplasma* Infection

**DOI:** 10.3389/fmicb.2021.666506

**Published:** 2021-06-17

**Authors:** Jinjin Zhu, Yang Wang, Yuanyuan Cao, Jilong Shen, Li Yu

**Affiliations:** The Key Laboratory of Microbiology and Parasitology of Anhui Province, The Key Laboratory of Zoonoses of High Institutions in Anhui, Department of Microbiology and Parasitology, School of Basic Medical Sciences, Anhui Medical University, Hefei, China

**Keywords:** *Toxoplasma gondii*, MIC1, MIC4, MIC6, invasion

## Abstract

*Toxoplasma gondii* microneme is a specialized secretory organelle that discharges its contents at the apical tip of this apicomplexan parasite in a sequential and regulated manner. Increasing number of studies on microneme proteins (MICs) have shown them as a predominant and important role in host cell attachment, invasion, motility and pathogenesis. In this review, we summarize the research advances in one of the most important MICs complexes, TgMIC1/4/6, which will contribute to improve the understanding of the molecular mechanism of *T. gondii* infection and provide a theoretical basis for the effective control against *T. gondii.*

## Introduction

*Toxoplasma gondii* is an obligate intracellular protozoan parasite of the phylum Apicomplexa with a unique apical complex composed of specialized cytoskeletal and secretory organelles, including rhoptries and micronemes. Microneme proteins (MICs) are known to be essential for the parasite invasion and adhesion to host cell (Carruthers and Sibley, 1997; Carruthers and Boothroyd, 2007; Santos and Soldati-Favre, 2011; Sibley, 2011). The expression of MICs is mostly in the form of functional complexes composed of adhesive and transmembrane domain-containing proteins, including TgMIC1/4/6, TgMIC3/8, TgMIC2/M2AP, and a complex of the apical membrane antigen 1 (TgAMA1) with rhoptry neck complex formed by *T. gondii* rhoptry neck protein (TgRON) 2, TgRON4, TgRON5, and TgRON8 (Cerede et al., 2005; Huynh and Carruthers, 2006; Sheiner et al., 2010; Besteiro et al., 2011; Lamarque et al., 2011; Checkley et al., 2015). Most MICs contain a series of structural domains such as thrombospondin type I-like repeat (TSR), apple-like, epidermal growth factor-like (EGF) and chitin binding-like (CBL) domains, etc. (Tomley and Soldati, 2001; Anantharaman et al., 2007) which share homology with higher eukaryotic proteins ligand domains or adhesive motifs. This might account for parasite’s ability to infect a wide range of host cell types (Sheiner et al., 2010; Boucher and Bosch, 2015). TgMIC1/4/6, the first found and the most extensively investigated microneme complex in *T. gondii*, is composed of soluble adhesion proteins TgMIC1, TgMIC4, and transmembrane escorter protein TgMIC6 (Brecht et al., 2001; Cerede et al., 2005; Saouros et al., 2005; Marchant et al., 2012; Paing and Tolia, 2014). More and more evidence show that TgMIC1/4/6 complex not only participates in the invasion of *T. gondii*, but also in the pathogenesis and immune escape of the parasite. This review aims to provide a more comprehensive understanding of the multiple roles of the TgMIC1/4/6 in *T. gondii* infection.

## Structure Characteristics and Secretory Regulation of TgMic1/4/6

**FIGURE 1:**
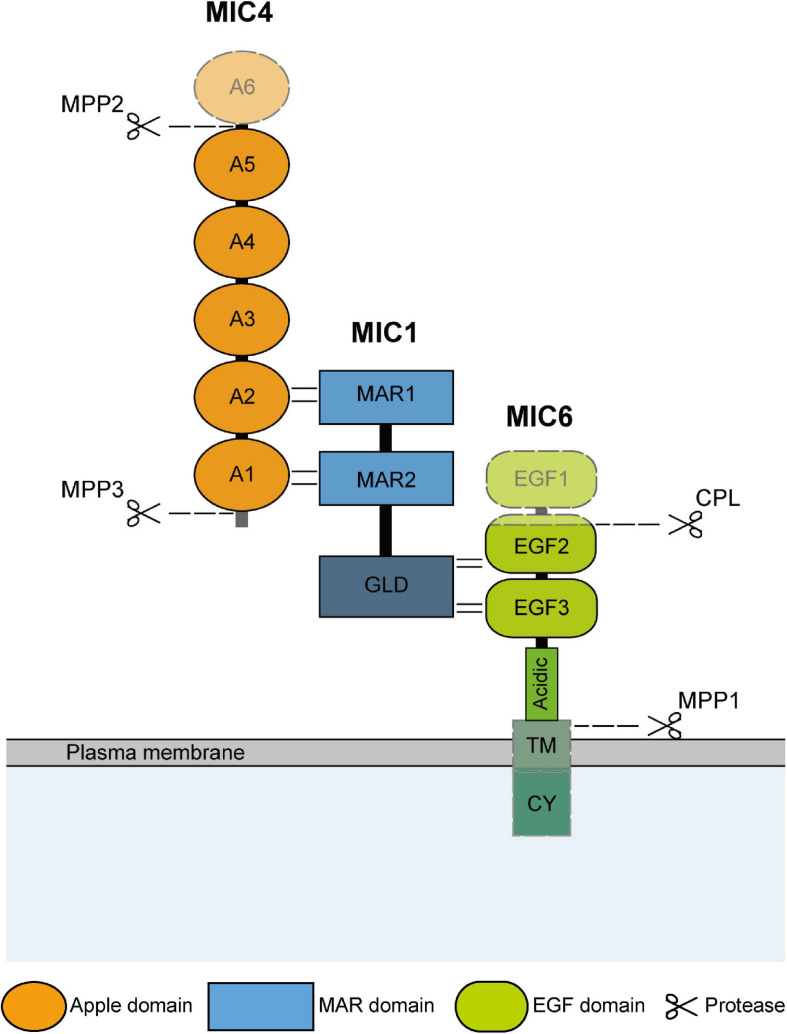
Schematic representation of domain structure and hydrolysis modification of TgMIC1/4/6. The domain structure and interaction sites of TgMIC1/4/6 are shown. And the C-terminal galectin-like domain (GLD) of MIC1, transmembrane (TM) and cytoplasmic domain (CY) of TgMIC6 are also indicated. TgMIC4 and TgMIC6 undergo hydrolysis modification by MPP2, MPP3, and CPL, respectively, during transport to the microneme, and then the complex is anchored on the surface of the parasites by TgMIC6, and finally released by MPP1 after the invasion is completed. The cleavage sites are all plotted.

TgMIC1 is one of the earliest identified MICs in *T. gondii*, which was obtained from tachyzoites by Fourmaux et al. (1996) through the monoclonal antibody screening in 1996. The *mic1* gene is 2,912 bp in length and contains three introns, and its open reading frame is 1,368 bp, encodes 456 amino acid residues with a predicted molecular mass (Mr) of 49 kDa. The N-terminal region of TgMIC1 possesses two micronemal adhesive repeat (MAR) domains formed by alternating stacked layers of tryptophan and arginine residues (Blumenschein et al., 2007; Garnett et al., 2009), which are certain homologous to thrombospondin 1 (TSP1) -like domain of thrombospondin-related anonymous proteins (TRAP) family in the *Plasmodium falciparum.* Importantly, the MAR domains have sialic acid lectin properties (Blumenschein et al., 2007; Garnett et al., 2009; Friedrich et al., 2010; Marchant et al., 2012), which can not only recruit TgMIC4 (Figure 1), but also interact with various sialic oligosaccharides on the surface of host cells. Analysis of the C-terminal structure of TgMIC1 revealed a non-functional galectin-like domain lacking sugar-binding residues, replaced by hydrophobic amino acids, which can interact with TgMIC6 and assist in folding and stabilizing the third EGF domain and its C-terminus extended acidic region (Saouros et al., 2005; Gay et al., 2014). Importantly, TgMIC1 is critical to ensure the successful exit of TgMIC1/4/6 from the early compartment of the secretory pathway, and helps the complex target the micronemes correctly. The absence of TgMIC1 leads to the accumulation of TgMIC4 and TgMIC6 in the perinuclear region, endoplasmic reticulum (ER) and Golgi (Di Cristina et al., 2000). Therefore, as a bridge for complex assembly, the knockout of the *mic1* gene alone is equivalent to the destruction of the complex overall function. Compared with the wild type, the invasion efficiency of TgMIC1 knockout strain was reduced by 50% (Cerede et al., 2005; Blumenschein et al., 2007; Friedrich et al., 2010).

TgMIC4 is distributed and approximately equal in the life stages of tachyzoites, bradyzoites, merozoites and oocysts of *T. gondii.* Its gene length is 1743 bp, containing 580 amino acid residues, and Mr is 61 kDa. TgMIC4 is composed of six apple domains, the apple domains 1 and 2, 3 and 4, and 5 and 6 are separated only by 3 amino acid residues, so they are pairing and the first and second domains referred to as MIC4-A12 (residues 67–230), the third and fourth ones as MIC4-A34 (residues 231–417), while the fifth and sixth ones as MIC4-A56 (residues 418–580) (Brecht et al., 2001; Brown et al., 2003; Coley et al., 2007; Dereeper et al., 2008; Gronwald et al., 2008). The MIC4-A12 domains directly interact with the two MAR domains of MIC1 (Reiss et al., 2001; Saouros et al., 2005, 2007). In *mic4 ko* strains, part of TgMIC1–6 complex was transferred to the micronemes, and only MIC4-A12 supplementation was sufficient to restore the excretion of TgMIC4 from the early secretory compartment and be sorted into the micronemes correctly. The functions of the MIC4-A34 domains are currently unclear. The A5 domain is a lectin with specificity for β1–3-or β1–4-galactosamine which is cleaved from the complex and is responsible for cell adhesion during parasite invasion (Yoshida et al., 2010; Marchant et al., 2012; Sardinha-Silva et al., 2019). TgMIC4 is initially synthesized in the form of a full-length 72-kDa and stored in micronemes. After discharge from the micronemes, microneme protein protease (MPP) 3 first cleaves the N-terminus of TgMIC4 into a 70-kDa species, followed by the release of 50- and 15-kDa fragments at the C-terminal through the MPP2 hydrolysis, exposing its galectin domain and promote tight binding to host cell receptors (Brecht et al., 2001; Brossier et al., 2005; Dowse et al., 2005; Figure 1).

TgMIC6 is a 34 kDa type I transmembrane protein that anchors the complex to the parasite membrane during the invasion. It contains three EGF-like domains (Keates et al., 2001; Meissner et al., 2002a; Koff et al., 2006), a transmembrane domain, and a carboxyl-terminal cytoplasmic domain (Reiss et al., 2001; Figure 1). The EGF domain is composed of 30–40 amino acid residues, contains six cysteine residues, which form three disulfide bonds, and is an evolutionarily conserved protein domain that widely presents in membrane-bound proteins and extracellular eukaryotic proteins, generally increases specificity through multivalent interaction and participates in many different biological functions (Davis, 1990; Li et al., 2015). A classification signal based on tyrosine residues in the C-terminal cytoplasmic domain of TgMIC6 is also critical for the correct transport of the complex to the micronemes. It is possible that the TgMIC1 galectin-like domain assists in the folding of the TgMIC6 C-terminal, so that the complex can exit from the ER and the Golgi, and then accurately transported into the microneme through the sorting signal (Reiss et al., 2001; Saouros et al., 2005). The knockout of TgMIC6 or its cytoplasmic region can cause TgMIC1 and TgMIC4 to remain in dense granules along the secretory pathway (Saouros et al., 2005). While TgMIC6 is transported into the trans-Golgi network (TGN) after synthesis, cathepsin L-like protease (CPL) is responsible for removing its first and partial second EGF-like domain (Figure 1; Reiss et al., 2001; Parussini et al., 2010). The role of this pre-domain removal is unclear, but it does not impact on the interaction between TgMIC6 and TgMIC1 (Parussini et al., 2010). After the complex is secreted outside the parasite, TgMIC6 is responsible for anchoring the complex on the surface, interacting with the actin-myosin system in the parasite, and gradually transfer to the back to provide power to penetrate the host. And then, rhomboid (ROM) 4, ROM5 and MPP1 hydrolyze the intramembrane region of the C-terminal transmembrane domain, so that the complex can be cleaved off the surface of *T. gondii* and disconnected from the host cell, resulting in an effective invasion.

**FIGURE 2:**
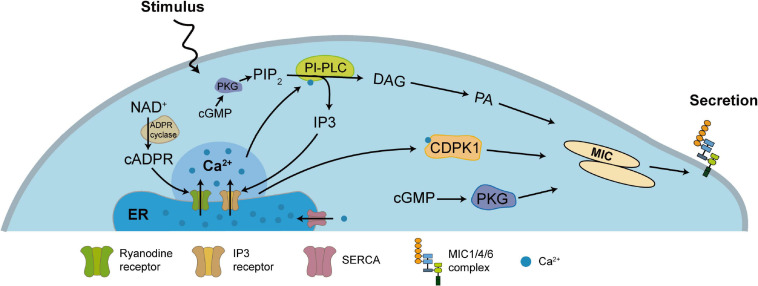
Ca^2+^-dependent secretory regulation pathway of TgMIC1/4/6. Most of Ca^2+^ in *T. gondii* are stored in the ER and produce a series of signaling cascades after stimulation. PIP_2_ generates IP3 and DAG through PLC, or NAD^+^ generates cADP-ribose through ADP ribosyl cyclase, and releases Ca^2+^ into the cytoplasm by the action of the Ca^2+^ channel on the ER membrane, which further converts DAG into phosphatidic acid (PA), and also activates CDPK1 to stimulate MICs secretion. Independently, activation of cGMP can activate PKG, which is also important for controlling MICs secretion. Other studies in the malaria parasite indicate that cGMP and PKG also indirectly control the synthesis of PIP_2_. On the other hand, SERCA pumps Ca^2+^ back into the lumen of the ER to restore the level of Ca^2+^ in the ER and cytoplasm.

After TgMIC1/4/6 undergo conformation-dependent sorting and proteolytic processing events to obtain functional integrity, the regulation of their secretion becomes a necessary condition for the invasion of *T. gondii* into host cells, and the level of calcium ions in the parasites plays a key role in this process. Under normal circumstances, TgMICs secretion occurs at extremely low levels (Carruthers et al., 1999; Carruthers and Sibley, 1999), but while the parasite contacts the host cell surface, it triggers a signal cascade, which stimulates the release of large amounts of calcium stored in the ER, mitochondria, and acidocalcisomes (Bonhomme et al., 1993; Miranda et al., 2010), thereby leading to the secretion and accumulation of numerous MICs on the parasites surface, mediating the gliding motility and invasion (Figure 2). Previous studies showed that inositol 1,4,5-trisphosphate (IP3) (Fang et al., 2006; Bullen and Soldati-Favre, 2016) and cyclic ADP ribose (cADPR) can mediate the release of intracellular Ca^2+^. These signaling molecules require secondary messengers such as phosphatidylinositol phospholipase C (PI-PLC) (Bullen and Soldati-Favre, 2016), cADPR cyclase and hydrolase to mediate Ca^2+^ release from the ER, however, there is no data on the existence of secondary messenger receptors (Nagamune et al., 2008; Jones et al., 2009; Singh et al., 2010). It has also been found that calcium-dependent protein kinase 1 (TgCDPK1) (Lourido et al., 2010, 2012; Lourido and Moreno, 2015; Brochet and Billker, 2016) and cyclic GMP (cGMP) activated protein kinase G (PKG) (Brown et al., 2017) which play a role in the downstream pathway of Ca^2+^, specifically regulating the secretion of MICs until the end of invasion, and after that within the host cell, *T. gondii* ER uptakes Ca^2+^ to store via SERCA-type Ca^2+^ -ATPases, in order to use in the process of egress and the next invasion (Figure 2; Billker et al., 2009; Lourido and Moreno, 2015). Ca^2+^-mobilizing agents such as calcium ionophores, thapsigargin, ethanol have been shown to stimulate the increase of intracellular calcium levels in *T. gondii* (Kawase et al., 2007). In recent years, there have also been reports that *T. gondii* can also use sex steroid hormones such as estradiol and progesterone to regulate intracellular Ca^2+^ signaling to promote infection and reproduction (Zhang et al., 2017, 2018). This may also be one of the reasons for the high transmission frequency and high incidence of *T. gondii* during pregnancy (Roberts et al., 2001).

## TgMIC1/4/6 Complex in the Invasion of *Toxoplasma*

**FIGURE 3:**
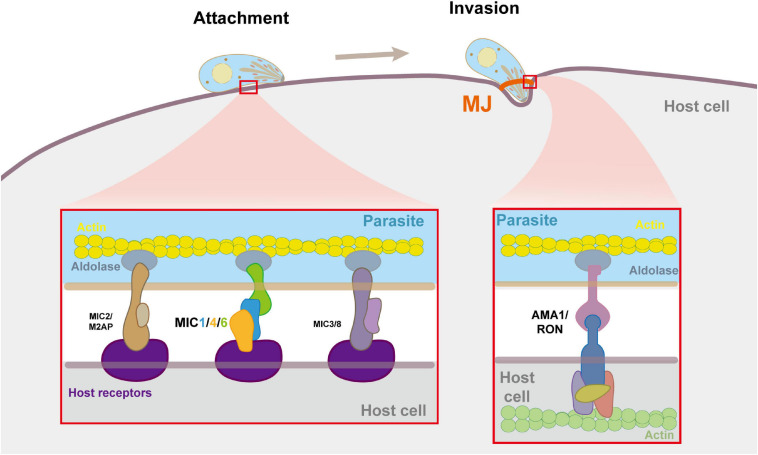
Molecular details of microneme complexes formated during host cell adhension and invasion by *T. gondii.* In the process of adhesion and invasion, MICs and RONs are secreted by the parasites, and form multiple multi-molecular complexes on the parasite and the host cell plasma membrane to bind them tightly, thereby triggering cell invasion. The enlarged image shows the detailed molecule interactions at the moving junction (MJ). MICs bind to receptors on the host cell surface. The AMA1-RON2 interaction forms MJ which anchors the parasite to the actin cytoskeleton of the host cell. Within the parasite pellicle, MICs bind to aldolase, which connects the complex to the parasite’s F-actin.

Unlike the mechanisms by which viruses and bacteria enter the cell through endocytosis or phagocytosis, *T. gondii* secreted a large amount of MICs during early contact of apical end with the host cell plasma membrane to establish connection with host cell receptors, and mediate gliding movement through interaction with the parasite actin-myosin system to invade host cells actively (Dobrowolski et al., 1997; Meissner et al., 2002b; Carruthers and Tomley, 2008; Soldati-Favre, 2008). It is generally believed that in the microneme protein complexes, adhesion proteins act as host cell receptor ligands, providing a “molecular bridge” for the combination of parasites and host cells, and transmembrane proteins establish a connection with the parasite actin-myosin system through their cytoplasmic tails, providing the power for penetration, and thus initiating an active invasion (Zheng et al., 2009; Figure 3). As the first such complex found in *T. gondii*, TgMIC1/4/6 is very typical.

Initially, Lourenço et al. (2001) first identified proteins from *T. gondii* with lectin activity, isolated MIC1 and MIC4 from the lactose-bound fraction (Lac+) by affinity chromatography on an immobilized lactose column and they bound to fetuin or human A erythrocyte in a way that can be inhibited by specific carbohydrate, and demonstrated that MIC1 is the lectin present in Lac+ responsible for the adsorption to immobilized lactose and for the hemagglutinating activity. But then it was found that MIC4 instead of MIC1 binds lactose. More precisely, the recognition of host cell surface carbohydrate structures in this complex mainly depends on the MAR domain of TgMIC1 and the A5 domain of TgMIC4, which specifically recognize α2-3-sialyllactosamine and β1-3- or β1-4-galactosamine, respectively (Friedrich et al., 2010; Marchant et al., 2012). Sialic acid widely exists in glycoproteins and glycolipids on the cell surface (Wang and Brand-Miller, 2003; Varki et al., 2009), plays an important role in the interaction of many viruses and bacteria with host cells, and is the vital determinant for *T. gondii* to recognize and invade host cells (Blumenschein et al., 2007; Takabatake et al., 2007; Persson et al., 2008; Friedrich et al., 2010; Baba et al., 2015). When using excess sialic acid or sialidase pre-incubated human fibroblasts, the tachyzoite showed an 85% reduction in invasion efficiency (Sugioka et al., 1987), with N-acetylneuraminic acid competitive inhibition or neuraminidase treatment of host cells, invasion can be suppressed by 90% (Blumenschein et al., 2007). Therefore, the high affinity of TgMIC1 and TgMIC4 to the side chain sialic acid moiety and terminal galactose moiety of gangliosides, which widely present in the cell membrane of neurons, may be an important factor in the tropism of *T. gondii* to the brain in the intermediate host (Karlsson, 1998). In addition, TgMIC1 may prevent the excretion of the parasite from the intestine after being ingested by binding to sialic acid on the gut epithelial cell wall (Cowper et al., 2012). Although the presence of TgMIC4 does not improve the host cell binding efficiency of TgMIC1 (Saouros et al., 2005), the high affinity and synergistic effects of TgMIC1, TgMIC4, and other microneme adhesion proteins on the surface receptors of different cell types may better explain the wide host range of *T. gondii*.

The tryptophan site in the cytoplasmic tail of TgMIC6 can interact with aldolase (Zheng et al., 2009; Boucher and Bosch, 2014), indirectly establishing a connection with the submembrane actin-myosin system in *T. gondii* (Figure 1), thereby driving the parasite to move in a spiral manner and actively penetrate the host cells to complete the invasion (Frenal and Soldati-Favre, 2013). Correspondingly, compared with the wild type, the invasion efficiency of *mic6ko* strains to host cells was reduced by about 50% (Sawmynaden et al., 2008), which was similar to the invasion of *mic1ko* strains. However, due to the, respectively, deletion of TgMIC1 and TgMIC6 all result in the unsatisfactory targeting effect of complexes on micronemes, this invasion defect can be attributed to the absence of TgMIC1/4/6 complex without higher accuracy, but it also better illustrates the importance of this complex in invasion.

## TgMIC1 and TgMIC4 Are the New Identified Sensors of TLR2/4 to Initiate the Innate Immunity

As mentioned above, people used to associate TgMIC1 and TgMIC4 with the host cell adhesion more closely; however, there is currently evidence that the interaction between TgMICs with host cell receptors can lead to signal transduction events. Previous studies by Lourenço et al. (2006) demonstrated that Lac^+^ subcomplex of *T. gondii* containing TgMIC1 and TgMIC4 can induce protective immunity against *T. gondii* via Th1-type immune response, thereby reducing tissue parasitism and increasing survival rate of *T. gondii*-infected mice that were immunized with Lac+ preparation. Using TgMIC1, TgMIC4 or TgMIC6 or combinations of them as vaccines, evaluated the immune response, and determine the protection against experimental toxoplasmosis in C57BL/6 mice. The results demonstrated clearly that these microneme proteins are potential vaccines against *T. gondii* because they prevented or diminished the detrimental effects of the infection (Pinzan et al., 2015). Recent studies have also demonstrated that TgMIC1, TgMIC4 and TgMIC6 are capable of inducing IFN-γ production from CD4^+^ and CD8^+^ effector memory T cells in mice chronically infected with *T. gondii*, further showing the role of TgMIC1 and TgMIC4 as immunomodulators (Saraav et al., 2019). In addition, an attenuated live strain of MIC1- and MIC3 genes-deleted *T. gondii* (Mic1–3KO) proved to be an effective vaccine candidate in mouse and sheep models (Ismael et al., 2006; Mevelec et al., 2010), with immunogenicity, well-tolerated, and safe to the felines, although it did not abolish the oocysts shedding after natural infection with wild-type *T. gondii* (Le Roux et al., 2020).

**FIGURE 4:**
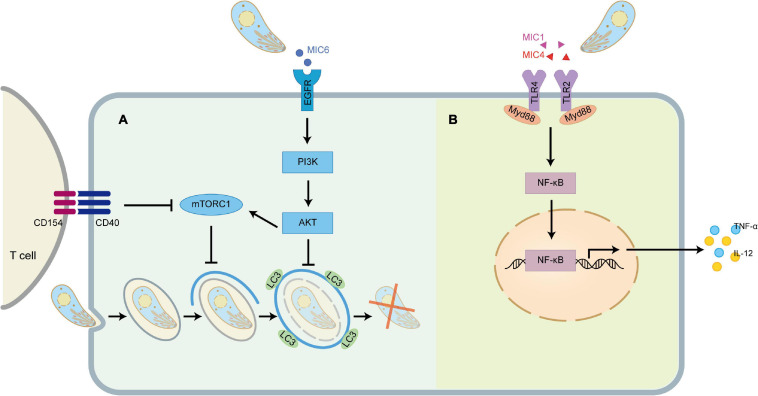
TgMIC1/4/6 activates host immunity and immune evasion. **(A)** TgMIC6 secreted by *T. gondii* can bind to EGFR on the cell surface, activate the PI3K/AKT signaling pathway, prevent the initiation of phagophore and impair the ability of CD154 to induce LC3 accumulation around the parasite. **(B)** TgMIC1 and TgMIC4 can interact with TLR2 and TLR4, triggering the Myd88-dependent NF-κB pathway to induce the production of IL-12 and TNF-α.

More significantly, researchers recently discovered that TgMIC1 and TgMIC4 are sensors of TLR2/4 to initiate the innate immunity (Sardinha-Silva et al., 2019). The extracellular leucine-rich repeat regions of TLR2 and TLR4 contain four and nine N-glycans, respectively. MIC1-MAR domain and MIC4-A5 domain can directly interact with the N-glycans of the extracellular domains of TLR2 and TLR4 expressed on macrophages and dendritic cells through non-classical carbohydrate recognition, triggering MyD88-dependent NF-κB pathway induces innate immune cells to produce IL-12, TNF-α and other pro-inflammatory cytokines, activate a protective immune response to produce resistance to parasites (Figure 4B). In mice infected with *T. gondii*, however, it is TgMIC1 instead of TgMIC4 that can cause systemic IFN-γ-induced level imbalance and pro-inflammatory cytokine storms, leading to acute death during the infection. Furthermore, they also found that in the response induced by TgMIC1/4, TLR2 heterodimerization with TLR1 or TLR6 and engagement of the co-receptors CD36 and CD14 enhanced the activation of cells, and proved that TgMIC1/4 up-regulates IL-12 through TGF-β activated kinase 1 (TAK1), p38 mitogen-activated protein kinase (p38), and NF-κB-dependent pathways (Mendonca-Natividade et al., 2019). Interestingly, in addition to inducing proinflammatory cytokine release by macrophages, MIC1 and MIC4 also trigger secretion of the anti-inflammatory cytokine IL-10 through an unknown mechanism in a TLR4 internalization-dependent manner. Meanwhile, the fact that the stimulated macrophages acquired transient tolerance to LPS provides a possible mechanism for evasion of the host inflammatory response by *T. gondii* (Ricci-Azevedo et al., 2021).

## TgMIC6 Helps the Parasite Escape From the Elimination of the Canonical Autophagy Pathway

Autophagy is a mechanism stimulated by innate and adaptive immune mechanisms, in which intracellular damaged or superfluous proteins and organelles are gradually surrounded by phagocytic vesicles with a double-layer membrane structure that subsequently forms autophagosomes, a structure that deliver their contents to the lysosomes for degradation (Mizushima et al., 2011). As we all know, *T. gondii* will form a parasitophorous vacuole (PV) that does not fuse with host cell lysosomes after invasion. Previous studies have shown that *T. gondii*-infected macrophages can pass through the CD40 on its surface and be activated. The binding of CD154 on the surface of CD4^+^ T cells activates the upstream regulators of autophagy, such as ULK1 and Beclin1-PI3KC3, which initiates the formation of phagophore and drives the accumulation of the autophagy protein LC3 on the phagophore, thereby eliminating the *T. gondii* hiding in the PV (Muniz-Feliciano et al., 2013). To avoid being targeted, one of the strategies developed by *T. gondii* is to inhibit autophagy by activating the epidermal growth factor receptor (EGFR)/Akt signaling pathway. EGFR is expressed in a variety of cell types, including retinal pigment epithelial cells, endothelial cells, microglia and macrophages (Sobolewska et al., 2009), and is an important driver of Akt activation by *T. gondii* (Muniz-Feliciano et al., 2013; Purba et al., 2017). MIC3, MIC6, MIC8 have multiple domains with homology to EGF (Meissner et al., 2002a), which act as EGFR ligands, bind to and induce autophosphorylation of the protein at tyrosine 1,148 in host cells, and induce rapid activation of Akt through PI3K (Cabodi et al., 2004). MIC1 ko (deficient in MIC6), MIC3 ko and especially MIC1/3 ko parasites are defective in induction of EGFR-Akt activation (Muniz-Feliciano et al., 2013). Although MIC8 has EGF-like domains, MIC8 ko parasites show no defect in EGFR-Akt activation (Muniz-Feliciano et al., 2013). The activation of EGFR-Akt in turn activates mammalian target of rapamycin 1 (mTORC1) which downregulates autophagosome formation via CD40-CD154-mediated autophagy pathway. In addition, activation of EGFR-Akt downregulates LC3 accumulation around the parasite to prevent the vacuole-lysosomal fusion, thus effectively preventing the killing of *T. gondii* (Figure 4A).

## Conclusion

*T. gondii* is considered to be one of the most successful parasites in the world, developing a series of strategies to fight against host defense. Based on the study of the multiple functions of TgMIC1/4/6 complex, it is speculated that the complex is one of the strategies for successful parasitism. This complex can not only help *T. gondii* to quickly “hide” into target cells, but also help the parasite “escape” immune clearance. However, MIC1 and MIC4 proteins in the complex were found to activate innate immunity and induce protective immunity against *T. gondii*. The inconsistency of functions of the complex may be explained the delicate balance between induction and suppression of host immune response, so as to ensure the host as a safe place for *T. gondii* survival and allow its transmission to the end host. The domain functions of TgMIC1/4/6 and some other MICs are duplicated. For example, both TgMIC1 and TgMIC13 have MAR domains, which can recognize sialic acid oligosaccharides; TgMIC6, TgMIC3, and TgMIC8 all have multiple domains homologous to EGF, etc. Why does *T. gondii* need such a variety of MICs and duplicate domains? Are their functions redundant? One of our speculations is that the diversity of this molecule may be to recognize a wider range of host receptors, coordinate the immune regulation of host cells, and ensure the efficient invasion and survival of *T. gondii* against a variety of host cells. This is effectively reflected in the up-regulated expression of AMA1 and RON2 homologs in *AMA1* knockout strains, which compensatively supported residual invasion (Lamarque et al., 2014). In addition, there is increasing evidence that TgMIC1, TgMIC4, and TgMIC6 are effective antigen targets and candidate vaccines, which can induce protective immunity against *T. gondii* through TH1 specific immune response. Nevertheless, there is still much work to be done on the study of MICs, and we believe that with the development of molecular biology and genetic engineering technology, more MIC complexes may be discovered, and further breakthroughs will be made in parasite-host interaction, signal recognition, structure and function.

## Author Contributions

LY and JZ designed the work. JZ and YW drafted the article and diagramming. YC, JS, and LY did critical revision of the article. All authors contributed to the article and approved the submitted version.

## Conflict of Interest

The authors declare that the research was conducted in the absence of any commercial or financial relationships that could be construed as a potential conflict of interest.
